# Factors contributing to subjective well‐being and supporting successful aging among rural Japanese community‐dwelling older adults: A cross‐sectional and longitudinal study

**DOI:** 10.1111/ggi.14835

**Published:** 2024-02-23

**Authors:** Kensaku Shojima, Takara Mori, Yosuke Wada, Hiroshi Kusunoki, Kayoko Tamaki, Ryota Matsuzawa, Koutatsu Nagai, Masashi Goto, Takahiro Tabuchi, Yasuyuki Nagasawa, Ken Shinmura

**Affiliations:** ^1^ Department of General Internal Medicine School of Medicine, Hyogo Medical University Nishinomiya Japan; ^2^ Cancer Control Center, Osaka International Cancer Institute Osaka Japan; ^3^ Amagasaki Medical COOP Honden Clinic Amagasaki Japan; ^4^ Roppou Clinic Toyooka Japan; ^5^ Department of Internal Medicine Osaka Dental University Hirakata Japan; ^6^ Department of Physical Therapy School of Rehabilitation, Hyogo Medical University Kobe Japan; ^7^ Department of General Medicine and Community Health Science Hyogo Medical University, Sasayama Medical Center Tambasasayama Japan

**Keywords:** functional capacity, Japan, older adults, subjective well‐being, successful aging

## Abstract

**Aim:**

We aimed to identify the factors contributing to subjective well‐being in community‐dwelling older adults in rural Japan. This study explored the relationship among physical and mental health, socioeconomic status, and activity levels with regard to the subjective well‐being of older adults.

**Methods:**

In the Frail Elderly in the Sasayama‐Tamba Area study, a cohort investigation of independent older adults in a rural Japanese community, 541 of 844 participants completed a 2‐year follow‐up survey. Subjective well‐being was assessed as a binary based on three factors – “happiness,” “satisfaction with life” and “meaning in life” – using a subset of the World Health Organization's Quality of Life questionnaire. The improvement group transitioned from not having subjective well‐being during the baseline survey to having subjective well‐being during the follow‐up survey. Furthermore, we used multivariable log‐Poisson regression models to calculate the prevalence ratios of subjective well‐being.

**Results:**

The cross‐sectional study showed that sleep satisfaction, health services access satisfaction and having a higher‐level functional capacity were positively associated with having “happiness” and “satisfaction with life.” Furthermore, being aged ≥80 years and having financial leeway were positively associated with having “meaning in life.” The longitudinal study showed that having a higher‐level functional capacity was positively associated with improving “happiness” and “satisfaction with life.” Being female was positively associated with improving “happiness” and “meaning in life,” and health services access satisfaction and alcohol drinking were positively associated with improving “satisfaction with life” and “meaning in life,” respectively.

**Conclusions:**

These findings offer promising avenues for enhancing the subjective well‐being of older adults. **Geriatr Gerontol Int 2024; 24: 311–319**.

## Graphical abstract

We evaluated the factors contributing to subjective well‐being, dividing it into “happiness,” “satisfaction with life” and “meaning in life” in Japanese rural community‐dwelling older adults. A cross‐sectional study showed that characteristics, such as sleep satisfaction, health services satisfaction and having a higher‐level functional capacity were associated with subjective well‐being. In contrast, a longitudinal study identified the same factors as well as different factors, such as being female, contributing to improving subjective well‐being.

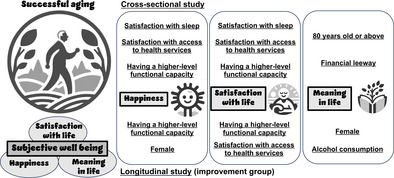

## Introduction

As the global population ages, the global community faces the pressing issue of creating a society where older individuals can enjoy healthy and vibrant lives based on successful aging. The aging process is intricately intertwined with physical, social and mental health; active participation in various facets of society; and the provision of protection, security and care.^1^ Successful aging is characterized by the absence of multimorbidity, robust functional capacity, active life participation and good health.^2^, ^3^ Subjective well‐being, referring to individuals' self‐reported assessment of their own well‐being, is crucial to achieving successful aging^4^ and has become increasingly critical in aging populations. A high subjective well‐being has a protective or positive effect on maintaining good health among older adults,^5^ and one meta‐analysis showed that high subjective well‐being is associated with decreased mortality risk.^6^

There has been no established method for measuring subjective well‐being since Diener first proposed the concept.^7^ Nevertheless, several distinct approaches that capture different aspects of subjective well‐being could be used. The following three elements are recommended at the minimum: hedonic well‐being, life evaluation and eudemonic well‐being.^5^ Hedonic well‐being refers to everyday feelings or moods, and one way to measure it is to ask respondents to rate their experience of “happiness.” Life evaluation refers to peoples' thoughts about the quality or goodness of their lives, and one way to measure it is to ask respondents to rate their experience of their “satisfaction with life.” Eudemonic well‐being focuses on judgments about the meaning and purpose of one's life, and one way to measure it is to ask respondents to rate their experience of “meaning in life.” Various scales have been developed to score and uniformly evaluate these elements, but they differ, and the factors that influence them are assumed to vary.

Exploring the subjective well‐being of rural communities in Japan is highly useful. In the face of increasing depopulation in large parts of Japan, the expansion of rural communities is becoming unavoidable.^8^ Amenities in rural areas are not as convenient as those in urban areas. Studies have shown, however, that in wealthy nations, rural living contributes to greater subjective well‐being than does urban living.^9^ In the Japanese language, the word ikigai is akin to eudemonic well‐being.^10^ A survey carried out by the Cabinet Office clearly showed that older people living in rural areas are more likely to have ikigai than those living in large cities.^11^ However, research on factors contributing to subjective well‐being in rural areas is lacking.

The primary objective of the present study was to elucidate the relationships among physical and mental health, socioeconomic status, and engagement in activities in regard to the three aspects of subjective well‐being among older adults residing in a rural community by their cross‐sectional relationships. In addition, we aimed to identify factors associated with changes in subjective well‐being based on longitudinal relationships. Enriching the subjective well‐being of older people is critical, but few studies have been carried out on this topic and dissected subjective well‐being into its facets. We attempted to fill that gap by investigating the factors contributing to subjective well‐being in older adults who lived in rural areas with the branch hospital of our university.

## Methods

### Study design and participants

This investigation had a prospective cohort design using data collected by the Frail Elderly in the Sasayama‐Tamba Area (FESTA) study, details of which can be found in our previous study.^12^, ^13^, ^14^ This research focused on individuals aged ≥65 years and residing in the Sasayama‐Tamba area, a rural region within Hyogo Prefecture. This region is characterized by rugged terrain, and a significant portion of its residents engage in farming. The area's population is 41 490, with an aging rate of 32.6%, contrasting with the 2015 urban Japan aging rate of 26.7%.

We carried out a baseline survey between September 2015 and December 2017. A 2‐year follow‐up survey was carried out between September 2017 and December 2019. Exclusion criteria comprised: (i) the presence of cognitive impairment, identified through a baseline assessment Mini‐Mental State Examination score of <21; and (ii) participants with incomplete data.

All procedures described in this work conform to the ethical standards of the relevant national and institutional committees on human experimentation and the Declaration of Helsinki. The Institutional Review Board of Hyogo Medical University reviewed and approved the protocol for this study (approval no. Rinhi0342). Participants received comprehensive oral and written explanations outlining the study's objectives, methodologies and anticipated outcomes before the survey's commencement. Before participation, all individuals provided written informed consent. The data used in this analysis were anonymized and masked to ensure privacy and confidentiality.

### Assessment of subjective well‐being and the possible factors related to it

The measure of subjective well‐being was based on items from the World Health Organization Quality of Life questionnaires.^15^, ^16^ “Happiness,” “satisfaction with life” and “meaning in life” were assessed using the following questions: “How much do you enjoy life?”, “How satisfied are you with yourself?” and “To what extent do you feel your life to be meaningful?”, respectively, ranked on a 5‐point response scale ranging from 1 (“not at all”) to 5 (“extremely”). Those who responded with a 4 or 5 to each question were considered to have “happiness,” “satisfaction with life” or “meaning in life,” accordingly.

We divided the participants into three groups: the improvement group (who went from not having subjective well‐being to having subjective well‐being), the unchanged group and the worsening group (who went from having subjective well‐being to not having subjective well‐being).

Characteristics of the sample group were sex, age group (65–69 years, 70–79 years or ≥80 years ), education (≤12 years or >12 years of education), subjective economic status (having or lacking financial leeway), alcohol consumption (drinker or non‐drinker), smoking (never, former or current) and the number of comorbidities. Other possible factors related to subjective well‐being included frailty (robust, prefrail or frail), satisfaction with access to health services (yes or no), having a higher‐level functional capacity (yes or no), satisfaction with sleep (yes or no) and step counts (≤6000 steps/day or >6000 steps/day). We selected these variables from existing literature reviews and medical perspectives.^1^, ^17^

We defined frailty phenotypes based on limitations in three or more of the following five conditions, assessed using the Cardiovascular Health Study: slow gait speed, weakness, exhaustion, reduced activity and weight loss.^18^ The frailty score, adjusted according to the Japanese version of the Cardiovascular Health Study, was determined by counting the applicable conditions. Individuals without these conditions were classified as robust, whereas those presenting with one or two conditions were classified as prefrail.^19^

Our assessment of higher‐level functional capacity used the Japan Science and Technology Agency Index of Competence, which originated from the Tokyo Metropolitan Institute of Gerontology Index of Competence.^20^ The Japan Science and Technology Agency Index of Competence encompasses 16 items categorized into four groups: use of new equipment, information‐gathering, daily life management and social involvement. A high score on this index signifies a favorable functional status. A score of ≥13 out of 16 was defined as “having a higher‐level functional capacity.”

Additional descriptions of each variable's measurements are shown in the Supplementary Methods in Data S1.

### Statistical analysis

Using a baseline survey, we explored factors contributing to subjective well‐being in a cross‐sectional study. In a longitudinal study, the outcome is improving or worsening each component of subjective well‐being, and we used a follow‐up survey to determine changes in subjective well‐being. Variables are from the baseline survey in both cross‐sectional and longitudinal studies. Because the outcomes were not rare, we used multivariable log‐Poisson regression models to calculate the prevalence ratios (PRs) for subjective well‐being, and the models converged. Percentages were shown with 95% confidence intervals (CIs) calculated using Wald's method. All analyses were carried out using SAS software version 9.3 (SAS Institute, Cary, NC, USA).

## Results

**Figure 1:**
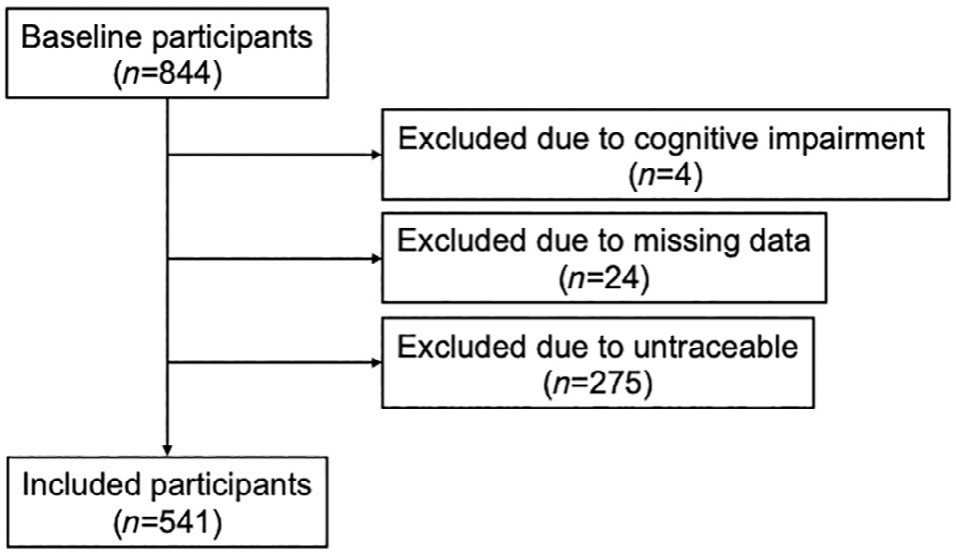
Flowchart showing the recruitment process and group allocation of the study participants.

In the initial phase of the survey, a baseline assessment was carried out on 844 older adults. As four individuals were excluded from the study due to cognitive impairment, and 24 due to missing data, the number of eligible participants in the baseline surveys was 816. Finally, 541 participants (64.1%) were tracked over 2 years, as 275 could not follow up (Fig. 1).

Among them, 352 were women, representing 65.1% of the group, whereas 189 were men, representing 34.9%. Of the total, 58.4%, 65.3% and 60.1% scored as having “happiness,” “satisfaction with life” and “meaning in life,” respectively (Table 1). Table 1 shows the PRs of well‐being for three aspects among all participants. The significant common factors associated with higher PRs for experiencing “happiness” and “satisfaction with life” than the reference category in the baseline survey were “satisfaction with sleep,” “satisfaction with access to health services” and “having a higher‐level functional capacity.” Furthermore, the significant factors linked to higher PRs for experiencing “meaning in life” than the reference category in the baseline survey were being aged ≥80 years and having financial leeway.

**Table 1:** Proportion and prevalence ratios of older adults' subjective well‐being based on the characteristics of study participants in the baseline survey

Factors contributing to happiness in older adults						
		*N*	*n*	% (95% CI)	PR^†^ (95% CI)	*P*‐value
Total		541	316	58.4 (54.3–62.6)		
Sex	Male	189	109	57.7 (50.6–64.7)	1 (Reference)	
	Female	352	207	58.8 (53.7–64.0)	1.15 (0.81–1.63)	0.432
Age group	65–69 years	224	115	51.3 (44.8–57.9)	1 (Reference)	
	70–79 years	257	160	62.3 (56.3–68.2)	1.17 (0.91–1.50)	0.226
	≥80 years	60	41	68.3 (56.6–80.1)	1.38 (0.94–2.00)	0.096
Education	≤12 years	429	249	58.0 (53.4–62.7)	1 (Reference)	
	>12 years	112	67	59.8 (50.7–68.9)	1.03 (0.78–1.37)	0.833
Economic status	No financial leeway	378	196	51.9 (46.8–56.9)	1 (Reference)	
	Financial leeway	163	120	73.6 (66.9–80.4)	1.25 (0.98–1.58)	0.067
Alcohol drinking	Non‐drinker	284	162	57.0 (51.3–62.8)	1 (Reference)	
	Drinker	257	154	59.9 (53.9–65.9)	1.15 (0.90–1.47)	0.273
Smoking	Never	388	232	59.8 (54.9–64.7)	1 (Reference)	
	Former	134	75	56.0 (47.6–64.4)	0.97 (0.68–1.38)	0.867
	Current	19	9	47.4 (24.9–69.8)	0.75 (0.37–1.53)	0.433
No. comorbidities	Nothing	113	69	61.1 (52.1–70.1)	1 (Reference)	
	1–2	358	220	61.5 (56.4–66.5)	1.00 (0.76–1.32)	0.998
	≥3	70	27	38.6 (27.2–50.0)	0.66 (0.42–1.04)	0.076
Frailty	Robust	243	161	66.3 (60.3–72.2)	1 (Reference)	
	Prefrail	278	148	53.2 (47.4–59.1)	0.89 (0.71–1.13)	0.344
	Frail	20	7	35.0 (14.1–55.9)	0.72 (0.33–1.57)	0.408
Satisfaction with access to health services	No	273	139	50.9 (45.0–56.9)	1 (Reference)	
	Yes	268	177	66.0 (60.4–71.7)	**1.27 (1.01–1.61)**	**0.042**
Having a higher‐level functional capacity	No	174	79	45.4 (38.0–52.8)	1 (Reference)	
	Yes	367	237	64.6 (59.7–69.5)	**1.31 (1.01–1.70)**	**0.045**
Satisfaction with sleep	No	220	98	44.6 (38.0–51.1)	1 (Reference)	
	Yes	321	218	67.9 (62.8–73.0)	**1.31 (1.01–1.71)**	**0.044**
Step counts	≤6000 steps/day	358	201	56.2 (51.0–61.3)	1 (Reference)	
	>6000 steps/day	183	115	62.8 (55.8–69.8)	1.10 (0.86–1.40)	0.453

Factors contributing to satisfaction with life in older adults						
		*N*	*n*	% (95% CI)	PR^†^ (95% CI)	*P*‐value
Total		541	353	65.3 (61.2–69.3)		
Sex	Male	189	130	68.8 (62.2–75.4)	1 (Reference)	
	Female	352	223	63.4 (58.3–68.4)	1.08 (0.78–1.51)	0.630
Age group	65–69 years	224	138	61.6 (55.3–67.9)	1 (Reference)	
	70–79 years	257	167	65.0 (59.2–70.8)	0.99 (0.78–1.26)	0.955
	≥80 years	60	48	80.0 (69.9–90.1)	1.32 (0.93–1.87)	0.117
Education	≤12 years	429	280	65.3 (60.8–69.8)	1 (Reference)	
	>12 years	112	73	65.2 (56.4–74.0)	0.93 (0.71–1.22)	0.618
Economic status	No financial leeway	378	225	59.5 (54.6–64.5)	1 (Reference)	
	Financial leeway	163	128	78.5 (72.2–84.8)	1.18 (0.94–1.48)	0.150
Alcohol drinking	Non‐drinker	284	176	62.0 (56.3–67.6)	1 (Reference)	
	Drinker	257	177	68.9 (63.2–74.5)	1.14 (0.91–1.45)	0.259
Smoking	Never	388	250	64.4 (59.7–69.2)	1 (Reference)	
	Former	134	90	67.2 (59.2–75.1)	1.03 (0.74–1.43)	0.868
	Current	19	13	68.4 (47.5–89.3)	0.98 (0.54–1.79)	0.957
No. comorbidities	Nothing	113	80	70.8 (62.4–79.2)	1 (Reference)	
	1–2	358	233	65.1 (60.2–70.0)	0.91 (0.70–1.18)	0.465
	≥3	70	40	57.1 (45.6–68.7)	0.84 (0.57–1.25)	0.393
Frailty	Robust	243	173	71.2 (65.5–76.9)	1 (Reference)	
	Prefrail	278	171	61.5 (55.8–67.2)	0.95 (0.76–1.18)	0.619
	Frail	20	9	45.0 (23.2–66.8)	0.75 (0.38–1.50)	0.419
Satisfaction with access to health services	No	273	150	55.0 (49.0–60.9)	1 (reference)	
	Yes	268	203	75.8 (70.6–80.9)	**1.34 (1.07–1.68)**	**0.010**
Having a higher‐level functional capacity	No	174	90	51.7 (44.3–59.2)	1 (reference)	
	Yes	367	263	71.7 (67.1–76.3)	**1.32 (1.03–1.69)**	**0.030**
Satisfaction with sleep	No	220	110	50.0 (43.4–55.6)	1 (reference)	
	Yes	321	243	75.7 (71.0–80.4)	**1.42 (1.13–1.79)**	**0.003**
Step counts	≤6000 steps/day	358	224	62.6 (57.6–67.6)	1 (reference)	
	>6000 steps/day	129	54	70.5 (63.9–77.1)	1.09 (0.87–1.37)	0.454

Factors contributing to meaning in life in older adults						
		*N*	*n*	% (95% CI)	PR^†^ (95% CI)	*P*‐value
Total		541	325	60.1 (56.0–64.2)		
Sex	Male	189	112	59.3 (52.3–66.5)	1 (Reference)	
	Female	352	213	60.5 (55.4–65.6)	1.15 (0.81–1.63)	0.448
Age group	65–69 years	224	116	51.8 (45.2–58.3)	1 (Reference)	
	70–79 years	257	164	63.8 (57.9–69.7)	1.20 (0.94–1.54)	0.121
	≥80 years	60	45	75.0 (64.0–86.0)	**1.51 (1.05–2.18)**	**0.026**
Education	≤12 years	429	255	59.4 (54.8–64.1)	1 (Reference)	
	>12 years	112	70	62.5 (53.5–71.5)	1.01 (0.77–1.34)	0.918
Economic status	No financial leeway	378	196	51.7 (46.7–56.7)	1 (Reference)	
	Financial leeway	163	129	79.1 (72.9–85.4)	**1.42 (1.13–1.79)**	**0.003**
Alcohol drinking	Non‐drinker	284	166	58.5 (52.7–64.2)	1 (Reference)	
	Drinker	257	159	62.2 (56.3–68.1)	1.14 (0.89–1.46)	0.286
Smoking	Never	388	237	60.6 (55.8–65.4)	1 (Reference)	
	Former	134	75	56.0 (47.6–64.4)	0.94 (0.66–1.34)	0.736
	Current	19	13	68.4 (47.5–89.3)	1.13 (0.62–2.07)	0.694
No. comorbidities	Nothing	113	69	61.1 (52.1–70.1)	1 (Reference)	
	1–2	358	220	61.5 (56.4–66.5)	0.98 (0.74–1.29)	0.873
	≥3	70	36	51.4 (39.7–63.1)	0.83 (0.55–1.26)	0.377
Frailty	Robust	243	157	64.6 (58.6–70.6)	1 (Reference)	
	Prefrail	278	157	56.5 (50.7–62.3)	0.92 (0.73–1.16)	0.495
	Frail	20	11	55.0 (33.2–76.8)	0.96 (0.51–1.81)	0.895
Satisfaction with access to health services	No	273	143	52.4 (46.5–58.3)	1 (Reference)	
	Yes	268	182	67.9 (62.3–73.5)	1.22 (0.97–1.53)	0.094
Having a higher‐level functional capacity	No	174	87	50.0 (42.6–57.4)	1 (Reference)	
	Yes	367	238	64.9 (60.0–69.7)	1.24 (0.96–1.60)	0.101
Satisfaction with sleep	No	225	121	54.1 (47.5–60.7)	1 (Reference)	
	Yes	321	206	64.2 (58.9–69.4)	1.08 (0.85–1.36)	0.527
Step counts	≤6000 steps/day	358	216	60.3 (55.3–65.4)	1 (Reference)	
	>6000 steps/day	183	109	59.6 (52.5–66.7)	1.00 (0.78–1.28)	0.989

^†^ Adjusted for all variables.

Boldface indicates statistical significance of *P* < 0.05.

CI, confidence interval; *N*, total number; *n*, number of participants showing subjective well‐being; PR, prevalence ratio.

Table 2 shows the changes in subjective well‐being during the 2‐year follow‐up period. In the follow‐up survey, carried out 2 years after the first, 233, 292 and 240 individuals who had “happiness,” “satisfaction with life” and “meaning in life,” respectively, in the baseline survey maintained the same status, whereas 83, 61 and 85 switched to not having “happiness,” “satisfaction with life” and “meaning in life,” respectively. Furthermore, 165, 128 and 150 individuals who did not have “happiness,” “satisfaction with life” and “meaning in life,” respectively, in the baseline survey maintained the same status, whereas 60, 60 and 66 transitioned to having “happiness,” “satisfaction with life” and “meaning in life,” respectively.

**Table 2:** Changes in the well‐being status of all participants

“Happiness”				
Baseline status	*n* (%)	Follow‐up status	*n* (%)	Category
Having “happiness”	316 (58.4)	Having “happiness”	233 (43.1)	Unchanged
		Not having “happiness”	83 (15.3)	Worsened
Not having “happiness”	225 (41.6)	Having “happiness”	60 (11.1)	Improved
		Not having “happiness”	165 (30.5)	Unchanged

“Satisfaction with life”				
Baseline status	*n* (%)	Follow‐up status	*n* (%)	Category
Having “satisfaction with life”	353 (65.2)	Having “satisfaction with life”	292 (54.0)	Unchanged
		Not having “satisfaction with life”	61 (11.3)	Worsened
Not having “satisfaction with life”	188 (34.8)	Having “satisfaction with life”	60 (11.1)	Improved
		Not having “satisfaction with life”	128 (23.7)	Unchanged

“Meaning in life”				
	*n* (%)	Follow‐up status	*n* (%)	Category
Having “meaning in life”	325 (60.1)	Having “meaning in life”	240 (44.4)	Unchanged
		Not having “meaning in life”	85 (15.7)	Worsened
Not having “meaning in life”	216 (39.9)	Having “meaning in life”	66 (12.2)	Improved
		Not having “meaning in life”	150 (27.7)	Unchanged

Table 3 presents the PRs of well‐being across three aspects in the group that experienced an improvement. The significant common factor associated with PRs for “happiness” and “satisfaction with life” that were higher than the reference category was “having a higher‐level functional capacity.” Being female was the common factor associated with PRs for “happiness” and “meaning in life” that were higher than the reference category. A significant factor linked to high PRs of experiencing “satisfaction with life” was “satisfaction with access to health services.” Furthermore, a significant factor linked to high PRs of experiencing “meaning in life” was alcohol consumption. By contrast, few factors were significantly associated with worsening subjective well‐being; the only negative predictor was “having a higher‐level functional capacity,” which negatively predicted “meaning in life” (Supplementary Table S1).

**Table 3:** Proportion and prevalence ratios of improved subjective well‐being among older adults based on the characteristics of study participants who did not have subjective well‐being in the baseline survey

Factors contributing to the improvement of happiness in older adults						
		*N*	*n*	% (95% CI)	PR^†^ (95% CI)	*P*‐value
Total		225	60	26.7 (20.9–32.4)		
Sex	Male	80	16	20.0 (11.2–28.8)	1 (Reference)	
	Female	145	44	30.3 (22.9–37.8)	**2.49 (1.03–6.01)**	**0.043**
Age group	65–69 years	109	31	28.4 (20.0–36.9)	1 (Reference)	
	70–79 years	97	26	26.8 (18.0–35.6)	1.10 (0.63–1.92)	0.746
	≥80 years	19	3	15.8 (0.0–32.2)	0.86 (0.24–3.15)	0.824
Education	≤12 years	180	50	27.8 (21.2–34.3)	1 (Reference)	
	>12 years	45	10	22.2 (10.1–34.4)	0.87 (0.43–1.77)	0.704
Economic status	No financial leeway	182	45	24.7 (18.5–31.0)	1 (Reference)	
	Financial leeway	43	15	34.9 (20.6–49.1)	1.45 (0.77–2.73)	0.244
Alcohol drinking	Non‐drinker	122	31	25.4 (17.7–33.1)	1 (Reference)	
	Drinker	103	29	28.2 (19.5–36.8)	1.25 (0.71–2.19)	0.437
Smoking	Never	156	41	26.3 (19.4–33.2)	1 (Reference)	
	Former	59	16	27.1 (15.8–38.5)	2.03 (0.91–4.54)	0.084
	Current	10	3	30.0 (1.6–58.4)	1.87 (0.46–7.50)	0.379
No. comorbidities	Nothing	44	12	27.3 (14.1–40.4)	1 (Reference)	
	1–2	138	37	26.8 (19.4–34.2)	1.07 (0.55–2.08)	0.850
	≥3	43	11	25.6 (12.5–38.6)	1.15 (0.48–2.77)	0.753
Frailty	Robust	82	20	24.4 (15.1–33.7)	1 (Reference)	
	Prefrail	130	37	28.5 (20.7–36.2)	1.42 (0.79–2.54)	0.242
	Frail	13	3	23.1 (0.2–46.0)	1.37 (0.36–5.16)	0.644
Satisfaction with access to health services	No	134	35	26.1 (18.7–33.6)	1 (Reference)	
	Yes	91	25	27.5 (18.3–36.6)	1.05 (0.59–1.88)	0.860
Having a higher‐level functional capacity	No	95	15	15.8 (8.5–23.1)	1 (Reference)	
	Yes	130	45	34.6 (26.4–42.8)	**2.15 (1.16–3.99)**	**0.016**
Satisfaction with sleep	No	122	29	23.8 (16.2–31.3)	1 (Reference)	
	Yes	103	31	30.1 (21.2–39.0)	1.36 (0.80–2.31)	0.255
Step counts	≤6000 steps/day	157	44	28.0 (21.0–35.1)	1 (Reference)	
	>6000 steps/day	68	16	23.5 (13.5–33.6)	0.89 (0.49–1.63)	0.707

Factors contributing to the improvement of satisfaction with life in older adults						
		*N*	*n*	% (95% CI)	PR^†^ (95% CI)	*P*‐value
Total		188	60	31.9 (25.3–38.6)		
Sex	Male	59	22	37.3 (25.0–49.6)	1 (Reference)	
	Female	129	38	29.5 (21.6–37.3)	0.76 (0.29–1.99)	0.570
Age group	65–69 years	86	27	31.4 (21.6–41.2)	1 (Reference)	
	70–79 years	90	31	34.4 (24.6–44.3)	0.99 (0.57–1.74)	0.984
	≥80 years	12	2	16.7 (0.0–37.8)	0.68 (0.14–3.21)	0.625
Education	≤12 years	149	47	31.5 (24.1–39.0)	1 (Reference)	
	>12 years	39	13	33.3 (18.5–48.1)	0.88 (0.46–1.71)	0.710
Economic status	No financial leeway	153	46	30.1 (22.8–37.3)	1 (Reference)	
	Financial leeway	35	14	40.0 (23.8–56.2)	1.02 (0.54–1.94)	0.943
Alcohol drinking	Non‐drinker	108	33	30.6 (21.9–39.2)	1 (Reference)	
	Drinker	80	27	33.8 (23.4–44.1)	0.80 (0.42–1.53)	0.498
Smoking	Never	138	41	29.7 (22.1–37.3)	1 (Reference)	
	Former	44	16	36.4 (22.2–50.6)	1.21 (0.51–2.84)	0.668
	Current	6	3	50.0 (10.0–90.0)	1.30 (0.29–5.88)	0.732
No. comorbidities	Nothing	33	8	24.2 (9.6–38.9)	1 (Reference)	
	1–2	125	43	34.4 (26.1–42.7)	1.59 (0.73–3.47)	0.247
	≥3	30	9	30.0 (13.6–46.4)	1.47 (0.54–3.97)	0.451
Frailty	Robust	70	29	41.4 (29.9–53.0)	1 (Reference)	
	Prefrail	107	27	25.2 (17.0–33.5)	0.63 (0.35–1.14)	0.128
	Frail	11	4	36.4 (7.9–64.8)	0.96 (0.31–3.03)	0.951
Satisfaction with access to health services	No	123	33	26.8 (19.0–34.7)	1 (Reference)	
	Yes	65	27	41.5 (29.6–53.5)	**1.90 (1.09–3.30)**	**0.024**
Having a higher‐level functional capacity	No	84	15	17.9 (9.7–26.1)	1 (Reference)	
	Yes	104	45	43.3 (33.8–52.8)	**2.51 (1.34–4.70)**	**0.004**
Satisfaction with sleep	No	110	30	27.3 (19.0–35.6)	1 (Reference)	
	Yes	78	30	38.5 (27.7–49.3)	1.33 (0.77–2.29)	0.306
Step counts	≤6000 steps/day	134	42	31.3 (23.5–39.2)	1 (Reference)	
	>6000 steps/day	54	18	33.3 (20.8–45.9)	0.89 (0.49–1.63)	0.713

Factors contributing to the improvement of meaning in life in older adults						
		*N*	*n*	% (95% CI)	PR^†^ (95% CI)	*P*‐value
Total		216	66	30.6 (24.4–36.7)		
Sex	Male	77	17	22.1 (12.8–31.3)	1 (Reference)	
	Female	139	49	35.3 (27.3–43.2)	**2.51 (1.02–6.17)**	**0.046**
Age group	65–69 years	108	38	35.2 (26.2–44.2)	1 (Reference)	
	70–79 years	93	24	25.8 (16.9–34.7)	0.85 (0.49–1.49)	0.574
	≥80 years	15	4	26.7 (4.3–49.1)	1.08 (0.35–3.41)	0.890
Education	≤12 years	174	53	30.5 (23.6–37.3)	1 (Reference)	
	>12 years	42	13	31.0 (17.0–44.9)	0.99 (0.50–1.97)	0.988
Economic status	No financial leeway	182	54	29.7 (23.0–36.3)	1 (Reference)	
	Financial leeway	34	12	35.3 (19.2–51.4)	1.06 (0.56–2.04)	0.852
Alcohol drinking	Non‐drinker	118	30	25.4 (17.6–33.3)	1 (reference)	
	Drinker	98	36	36.7 (27.2–46.3)	**1.82 (1.06–3.11)**	**0.029**
Smoking	Never	151	50	33.1 (25.6–40.6)	1 (Reference)	
	Former	59	15	25.4 (14.3–36.5)	1.24 (0.54–2.85)	0.618
	Current	6	1	16.7 (0.0–46.5)	0.75 (0.09–6.51)	0.797
No. comorbidities	Nothing	44	19	43.2 (28.6–57.8)	1 (Reference)	
	1–2	138	35	25.4 (18.1–32.6)	0.59 (0.38–1.26)	0.225
	≥3	34	12	35.3 (19.2–51.4)	0.96 (0.42–2.19)	0.929
Frailty	Robust	86	31	36.1 (25.9–46.2)	1 (Reference)	
	Prefrail	121	32	26.5 (18.6–34.3)	0.84 (0.48–1.46)	0.534
	Frail	9	3	33.3 (2.5–64.1)	0.96 (0.25–3.72)	0.952
Satisfaction with access to health services	No	130	36	27.7 (20.0–35.4)	1 (Reference)	
	Yes	86	30	34.9 (24.8–45.0)	1.51 (0.89–2.58)	0.128
Having a higher‐level functional capacity	No	87	23	26.4 (17.2–35.7)	1 (Reference)	
	Yes	129	43	33.3 (25.2–41.5)	1.15 (0.68–1.96)	0.604
Satisfaction with sleep	No	101	28	27.7 (19.0–36.5)	1 (Reference)	
	Yes	115	38	33.0 (24.5–41.6)	1.30 (0.76–2.23)	0.341
Step counts	≤6000 steps/day	142	41	28.9 (21.4–36.3)	1 (Reference)	
	>6000 steps/day	74	25	33.8 (23.0–44.6)	1.40 (0.82–2.40)	0.215

Boldface indicates statistical significance of *P* < 0.05.

CI, confidence interval; *N*, number of participants who did not have meaning in life in the baseline survey; *n*, number of participants with improving changes in subjective well‐being in the follow‐up survey; PR, prevalence ratio.

^†^ Adjusted for all variables.

## Discussion

The present cross‐sectional study showed common factors that were positively correlated with both “happiness” and “satisfaction with life,” and other factors were positively correlated with “meaning in life.”

Satisfaction with sleep was positively correlated with both “happiness” and “satisfaction with life.” Insomnia is prevalent in older adults, and a survey carried out of the general adult population in Japan found that 29.5% of individuals aged ≥60 years reported experiencing it.^21^ Furthermore, subjective sleep sufficiency is strongly associated with several health conditions, including subjective well‐being.^22^ Although investigating the relationship between objective sleep quality and subjective well‐being remains a challenge, improving satisfaction with sleep might be important to older adults' subjective well‐being.

Satisfaction with access to health services and having a higher‐level functional capacity were also associated with “happiness” and “satisfaction with life.” Previous studies in China have shown that access to health services is significantly associated with life satisfaction.^23^ Although Japan is considered to have relatively good access to medical care, the rural setting of this study might have influenced our results. In contrast, a previous paper from Sweden reported that a decline in activities of daily living leads to lower life satisfaction among older adults.^24^ The results of our study, in which having a higher‐level functional capacity is significantly related to the life satisfaction of older adults, are convincing.

It is fascinating to note that, unlike the other aspects of subjective well‐being, being aged ≥80 years and having financial leeway were associated with having a sense of “meaning in life.” It is assumed that older people tend to have a more positive outlook on life.^25^ Furthermore, previous reports have shown that having financial leeway is associated with having “meaning in life” in older adults.^26^ Having “meaning in life” is a critical aspect of the subjective well‐being of older adults.^27^

The status of subjective well‐being is not necessarily universal, and it changed during the 2‐year follow‐up period. Longitudinal study identified several characteristics of those likely to improve their subjective well‐being during the follow‐up period.

In the present study, having a higher‐level functional capacity was generally found to be associated with subjective well‐being. Historically, a decrease in functional capacity has been associated with reduced subjective well‐being.^28^ Frailty was found to be associated with declining levels of well‐being among community‐dwelling older people in a Canadian study.^29^ Although few studies in Japan have considered frailty in the context of subjective well‐being, it is essential to examine it when investigating subjective well‐being. The present study, however, did not identify a correlation between subjective well‐being and the state of frailty, even though the percentage of people with subjective well‐being had a corresponding decrease in their frailty. Having a higher‐level functional capacity might have been a confounding factor in this respect.

In terms of having “happiness” or “meaning in life,” it is interesting that being female was a predictor of improvement in these areas. Although there are few differences in subjective well‐being based on sex,^30^ this observation might indicate that women are more adaptable than men. Satisfaction with access to health services also predicted improvement in having “satisfaction with life.” Access to healthcare is essential for older adults as social capital and might positively modify their subjective well‐being, which can fluctuate.

Alcohol consumption might positively or negatively affect an individual's well‐being. The effects of alcohol can vary based on different factors, such as the amount consumed, frequency of consumption, individual lifestyle and health condition. This study did not investigate the amount of alcohol consumed. Excessive alcohol consumption causes multiple diseases.^31^ Appropriate alcohol consumption, however, might positively affect social relationships in a rural community.^32^ Further investigations are needed.

The ability of older adults to maintain their physical activity levels, and continue engaging in social, economic, cultural, spiritual and civic pursuits signifies active and healthy aging.^33^ Furthermore, vibrant, healthy aging involves maximizing opportunities to achieve successful aging.^1^ In this context, subjective well‐being and its associated individual physical aspects, and community and social factors can serve as potent instruments in the pursuit of successful aging. The findings of this study could have implications for policies to enhance the subjective well‐being, which in turn supports the successful aging, of older adults in Japan.

There were several limitations in the present study. First, it might be subject to participant selection bias, because it relied on older individuals who voluntarily engaged in medical checkups. Participants were recruited through community newspaper advertisements or oral announcements, which could have introduced bias, as those who responded tended to be health‐conscious and in relatively good health. Consequently, the study had a low proportion of frail participants. Second, although we screened for extreme responses, the data collected were based on self‐reports and lacked systematic validation testing, potentially affecting the accuracy of the information gathered. Third, the follow‐up rate of 64% was relatively low, partly due to the location of the Tamba‐Sasayama area, which is in a rural part of Hyogo Prefecture and has limited access to public transportation. Consequently, many participants, especially those experiencing a decline in physical ability, might have been unable to attend the follow‐up survey, resulting in potential dropout bias. Supplementary Table S2 provides a detailed overview of the study participants, distinguishing between those who were followed up after 2 years and those who were not. A significantly higher percentage of participants who could not be followed up were those who lacked financial leeway, were current smokers, non‐drinkers or frail, or had “a higher‐level functional capacity.” This result indicates which attributes are missing due to dropping out. Finally, it might be more desirable to construct a novel validated method of assessing subjective well‐being that does not use a single question on the World Health Organization Quality of Life questionnaire to evaluate hedonic well‐being, life evaluation and eudemonic well‐being, but rather, a more accurate assessment. Quality of life and subjective well‐being are closely related, but distinct concepts. Assessing only quality of life does not capture all the facets of subjective well‐being. Therefore, although assessing subjective well‐being in three aspects was one of the solutions to evaluate the subjective well‐being of older adults, it is essential to recognize its limitations and integrate other assessment methods or approaches in the future to have a comprehensive understanding.

The present study examined subjective well‐being, dividing it into “happiness,” “satisfaction with life” and “meaning in life.” Multiple factors related to subjective well‐being, such as having a higher‐level functional capacity, were identified. Subjective well‐being did not consistently remain static, but changed throughout the 2‐year follow‐up period. Several factors, such as being female, were associated with this change. The findings show that the factors associated with subjective well‐being are likely to contribute to successful aging. Individual health and longevity are also crucial for building a healthy super‐aged society in the future. This study will help shape future policies and strategies for improving subjective well‐being.

## Disclosure statement

The authors declare no conflict of interest.

## Author contributions

KSho and KShi contributed to data curation, investigation, formal analysis, funding acquisition, writing of the original draft, and review and editing of the draft. TM, YW, HK, KT, RM, KN, MG and YN contributed to data curation, investigation, and review and editing of the draft. TT contributed to the investigation, and review and editing of the draft.

## Supporting information


**Data S1.** Supporting information.

## Data Availability

The data that support the findings of this study are available on request from the corresponding author. The data are not publicly available due to privacy or ethical restrictions.
