# Supplementary material for: Factors contributing to subjective well‐being and supporting successful aging among rural Japanese community‐dwelling older adults: A cross‐sectional and longitudinal study
Source: Geriatr Gerontol Int. 2024 Feb 23;24(Suppl 1):311–9. doi: 10.1111/ggi.14835 (PMC11503561; doi:10.1111/ggi.14835)
Supplement: Supplementary file 1 — Data S1. Supporting information. [file GGI-24-311-s001.docx]

**Supplementary Methods**

**Additional description of characteristics of the sample group**

The following items were evaluated using a questionnaire: sex, age, education, subjective economic status, alcohol drinking behavior, smoking behavior, number of comorbidities, satisfaction with access to health services, and satisfaction with sleep.

We tracked the walking steps of participants using a wrist-worn accelerometer (Actiband from TDK Co. in Tokyo, Japan). This device has shown a moderate correlation to hip-worn accelerometers (r=0.55–0.86).^1^ The wrist-worn accelerometers proved highly accurate in distinguishing between sedentary and other activities.^2^ The accelerometer operated in five-minute periods. Participants were instructed to wear the accelerometer constantly, including during sleep; they could remove it during bathing, however, if desired. Participants were not told about their physical activity feedback during the assessment. We calculated the numbers for those diligent participants who clocked in at least ten hours a day for a minimum of four days. A previous study approved the device's reliability and validity.^3^ There is no consensus on how many steps are appropriate for adequate exercise, but based on reports that walking 6,000-8,000 steps daily lowered mortality in those over age 60,^4^ this study used 6,000 or more steps as the cutoff.

Specific diseases that participants were asked about as comorbidities in this study included hypertension, diabetes mellitus, dyslipidemia, chronic kidney disease, hepatic disease, cardiovascular disease, asthma, tuberculosis, pneumonia, osteoporosis, rheumatoid arthritis, collagen disease, stroke, esophagogastric disease, thyroid disease, blood disease and history of various types of cancer.

**References**

1 Kamada M, Shiroma EJ, Harris TB, Lee I-M. Comparison of physical activity assessed using hip- and wrist-worn accelerometers. Gait Posture 2016; **44**: 23–8.

2 Mannini A, Intille SS, Rosenberger M, Sabatini AM, Haskell W. Activity Recognition Using a Single Accelerometer Placed at the Wrist or Ankle. Med Sci Sports Exerc 2013; **45**: 2193–203.

3 Nagai K, Tamaki K, Kusunoki H, Wada Y, Tsuji S, Itoh M, et al. Isotemporal substitution of sedentary time with physical activity and its associations with frailty status. Clin Interv Aging 2018; **Volume 13**: 1831–6.

4 Paluch AE, Bajpai S, Bassett DR, Carnethon MR, Ekelund U, Evenson KR, et al. Daily steps and all-cause mortality: a meta-analysis of 15 international cohorts. Lancet Public Health 2022; **7**: e219–28.

***Supplementary Table1:*** *Proportion and PRs of* worsened *subjective well-being among older adults based on the characteristics of study subjects who have subjective well-being in the initial survey*

*(1) Factors contributing to the worsening of happiness in older adults*

*(2) Factors contributing to the worsening of satisfaction with life in older adults*

*(3) Factors contributing to the worsening of meaning in life in older adults*

***Supplementary Table2:*** *Proportion of the baseline characteristics of analytic and non-analytic participants*
